# Sudden Infant Death Syndrome, Infection, Prone Sleep Position, and Vagal Neuroimmunology

**DOI:** 10.3389/fped.2017.00223

**Published:** 2017-11-14

**Authors:** Paul Nathan Goldwater

**Affiliations:** ^1^Discipline of Paediatrics, School of Paediatrics and Reproductive Health, University of Adelaide, North Adelaide, SA, Australia

**Keywords:** infection, sudden infant death syndrome, vagus, neuroimmunology, prone sleep position

## Abstract

Recent findings suggest that infection (and sepsis) stand alone as the only plausible mechanism of causation of sudden infant death syndrome (SIDS) and accordingly achieves congruence with all clinicopathological and epidemiological findings. This review examines the role of infection in the pathogenesis of SIDS in the context of the major risk factor of prone sleep position. The study explores how sleep position could interact with the immune system and inflammatory response *via* vagal neural connections, which could play key roles in gut and immune homeostasis. A plausible and congruent clinicopathological and epidemiological paradigm is suggested.

## Introduction

In a recent review of the role of infection in sudden infant death syndrome (SIDS) (1), it was demonstrated that approaches to SIDS research that failed to take into account the clinicopathology and epidemiology of the condition rendered these mainstream lines of research fundamentally flawed. The onus on future research, to maintain scientific relevance, therefore must be stringent in accounting for the aforementioned points. Without addressing these fundamentals, mainstream research will continue to provide largely irrelevant findings.

This study considers the role of infection in the etiopathogenesis of SIDS. Of all areas of SIDS etiological research, infection remains the only one that is completely congruent with the clinicopathological and epidemiological findings (1). Previously, it was shown that mainstream research (e.g., cardiorespiratory control) does not address these aspects in any specific way. Cases used in mainstream studies have little or no data pertaining to their pathological findings and/or epidemiological (risk) factors and leave the findings seriously wanting. Moreover, physiological explanations for a causal link between prone sleeping and sudden unexpected/unexplained deaths in infants (SUDI) remain unsatisfactory on several levels (1).

As part of the infection hypothesis (1–11), we and others have examined normally sterile sites at autopsy and have found potentially pathogenic bacteria (e.g., *Staphylococcus aureus* and coliforms) in a notable number of SIDS cases (12–14). No evidence of such bacteremia was found in sudden traumatic deaths serving as controls (13). It was considered the positive sterile site findings in SIDS cases possibly represented a “footprint” of a fatal bacteremic episode in the last hours of life. Postmortem translocation of bacteria, as a possible alternative explanation, is less likely given that sterile sites remain sterile in cases of traumatic deaths (*vide infra*). Potential source of infection in SIDS could logically arise from either the gut or the respiratory tract. In favor of a gut origin was the finding of non-Shiga toxin verotoxigenic *Escherichia coli* belonging to a restricted range of serogroups not seen in live matched control babies (15). In addition, these *E. coli* were capable of causing death in neonatal mouse pups (4). These studies led us to explore the role of the gut microbiome in SIDS, which gave us interesting preliminary findings (16). This work has expanded recently with the use of 16S RNA metagenomic analysis. This has shown no major differences between the two groups (unpublished data, submitted for publication). It should be noted that, however, as a consequence of its design, the metagenomic analysis was incapable of assessing *E. coli* serogroups or atypical toxigenic factors.

In epidemiological studies of SIDS, cesarean section (CS) has been shown not to be a SIDS risk factor (17, 18). While CS carries adverse developmental effects on the infant it does not appear to affect maturation and diversity of the infant microbiome (19) and such finding is congruent with the aforementioned lack of association of CS with SIDS.

The other possible origin of a hypothesized fatal bacteremic episode is the respiratory tract. Evidence supporting this includes inflammatory changes in the lungs and airways in SIDS babies (20, 21) and the high rate of *E. coli*/coliforms in lung tissue (12, 13). *S. aureus* is also a key player whose involvement as a respiratory pathogen in SIDS babies has been well documented (22). Further evidence of respiratory infection causing deaths akin to and mistaken for SIDS and which exhibit SIDS-like pathology (subpleural and subepicardial hemorrhages) is described (23). The described SIDS-like hemorrhages occurred in cases of overt interstitial pneumonia and acute bronchiolitis. Unfortunately, the authors failed to mention whether or not these cases also exhibited liquid unclotted blood (seen in all cases of SIDS); however, many of the cases had congested organs characteristic of SIDS. Upon enquiry, most of the proven interstitial pneumonia and bronchiolitis cases also exhibited subserosal petechial hemorrhages, liquid blood and edematous heavy brains characteristic of SIDS (Szollosi, personal communication). This series of cases establishes the fact that SIDS-like pathology occurs in pathologically recognizable respiratory infection in babies younger than a year. It is reasonable to conjecture that such infection results in overwhelming sepsis causing sudden unexpected death and that it is therefore logical that the same or similar underlying process (sepsis) is responsible for causing the typical pathological findings (24) in SIDS cases. On carrying this logic to its conclusion, we are bound to judge that overwhelming infection is the cause of the majority of SUDI.

## The Prone Sleep Position Risk Factor, Neuroimmunology, and the Role of the Vagus Nerve

A further clue in regard to the role of infection especially in relation to prone sleeping was revealed in a landmark study by Ponsonby et al. (25). The paper stated “The prone position increased the risk of SIDS more than 10-fold among ill infants, but it was associated with only a slight increase in risk among well infants. This difference in risk was significant (*P* = 0.02).” It should not be forgotten that many SIDS cases have died in supine or lateral positions. The underlying reasons for the increased risk of prone sleeping in babies undergoing an overt infectious process requires deeper enquiry and discussion (*vide infra*).

While our previous publications (1, 16, 26) proposed a plausible mechanism to account for the key risk factor of prone sleep position and which incorporated the idea of ingestion or inhalation of bacteria that contaminate the sleeping surface, supported by evidence showing such bacterially contaminated sleeping surfaces (parental bed, sofas, and previously used cot mattress) are proven risk factors for SIDS (27–30), there is a need to seek out additional and congruent physiological explanations to expand our knowledge and further explain the prone sleep risk factor. One of these might be the role of the vagus nerve, in particular in relation to interaction with the immune system and the gut microbiota.

Understanding of the role of the sympathetic and parasympathetic nervous systems in relation to immunological status has increased in recent times. Neurotransmitters such as noradrenaline, acetylcholine, histamine, vasoactive intestinal peptide, and substance P demonstrably play important immunological roles (31). In addition, cytokine regulation involves neuroendocrine hormones including corticotrophin releasing factor, melanocyte stimulating hormone, and leptin (31). Not only do interactions between the nervous and immune systems represent important homeostatic functions, but there is another major system participating in these functions whose importance is being revealed; and that is the role of the gut bacterial microbiota (32).

The two-way communication between the gut microbiota and the brain is essential in immune homeostasis and the main connection between brain and gut (and spleen) (the brain–gut axis) involves the vagus nerve. The gut bacterial microbiota provides important peptides and short-chain fatty acids that direct the development and maintenance of a healthy immune system (33). It is the combination of gut bacteria, diet, and prone sleep position and the neurophysiology of the vagus nerve (particularly during an infectious process) that this paper explores.

It has been shown in an animal model that neurochemical and other effects are abolished by vagotomy showing that the vagus acts as the major modulatory constitutive communication pathway between the gut microbiota and the brain (34). The gut microbiota also plays a role in neuroendocrine homeostasis (35).

Over the last decade, our understanding of the physiology and immunology of the cholinergic anti-inflammatory pathway (CAP) has expanded. Cytokines are key contributors to health and disease. Their production *via* the vagus nerve inflammatory reflex can prevent cytokine-induced tissue damage and death. Vagal stimulation has been shown in animal models to prevent cytokine release and damage during sepsis, endotoxemia, ischemia/reperfusion injury, and hypotensive shock (36). Moreover, vagal innervation of the gastrointestinal tract has been shown to play a key role in modulating intestinal immune activation. Electrical stimulation of the vagus profoundly inhibits intestinal inflammation normalizing intestinal homeostasis, whereas vagotomy has the reverse effect (37). Indeed, following infradiaphragmatic vagotomy of mice, stimulated T cells (CD4^+^) from the spleen produced increased levels of pro-inflammatory cytokines, e.g., TNF and IFN-gamma. Following administration of nicotine and the treatment of non-vagotomized animals with a nicotinic receptor antagonist, control levels of cellular responses were restored, mimicking the effect of vagotomy (38).

The question remains in regard to prone positioning: how does this affect vagal neurophysiology in the context of SIDS? A possible clue comes from a study of familial pattern overactivity of the vagus nerve indicating a possible link with risk of sudden death (39). Involvement of the sympatheticovagal pathway(s) is a logical area to explore. A study in infants showed prone sleeping did not significantly impact on heart rate variability (HRV) in preterm infants. However, reduced maturation of high frequency HRV in very preterm infants resulted in significantly altered sympathovagal balance at 2–3 months corrected age, the peak age of SIDS risk. The authors concluded this may contribute to the increased risk of SIDS in infants born at earlier gestational age (40).

A study of the effect of prone positioning in adult patients with acute respiratory distress syndrome also provides a clue. The levels of plasma IL-6 concentration declined significantly with time in the prone position group (*p* = 0.011) and predicted mortality assessed at day 14 (41). Similarly in adults, marked differences in vagal responses (measuring spectral HRV analysis) were observed between prone and supine positions (42).

Mainstream research has rightly postulated that central nervous system mechanisms (together with prone sleep position) contribute to SUDI/SIDS but for the wrong reasons. These include prolonged breath holding, failure of arousal, and laryngeal reflex apnea potentiated by upper airway infection and failure of brainstem-mediated autoresuscitation (43). All of these postulated mechanisms have been shown to lack congruency with pathological and epidemiological findings (1) and therefore cannot be seriously considered as valid areas of investigation. Therefore, it is important to explore other areas that adhere to the principles of congruency with pathology and epidemiology in SUDI/SIDS. Indeed, there seems to be only one: infection.

While no studies were found reporting positional vagal effects on immune function in babies, there are clues from animal studies that indicate a CAP in which cholinergic agonists inhibit cytokine production. Stimulation of the vagus prevents the untoward effects of cytokine release in experimental sepsis and endotoxemia (36). It is thought that the CAP and immune homeostasis are mediated through the vagal innervation of the gastrointestinal tract (37). Non-vagal spinal pathways may also be involved in the anti-inflammatory response. An alternative pathway involving C1 neurones located in the medulla oblongata mediates adaptive autonomic responses to physical stressors (including exposure to bacterial lipopolysaccharides) possibly *via* spinal neurones (44).

Whether or not prone body position cancels out anti-inflammatory pathways remains to be elucidated. Other factors such as nicotine exposure may play a role; Blood-Siegfried et al. (45) found that rat pups die from a combination of infectious insults during a critical time of development. This is exacerbated by perinatal nicotine exposure. Such exposure was shown to alter the autonomic responses in exposed offspring. Others have shown effects on vagal tone and inflammation through exposure to smoke (46). Exposure to smoke is a known risk factor for SIDS (1).

## Conclusion

Clinicopathological and epidemiological evidence suggests that infection is the underlying basis for most sudden unexpected deaths in infancy. Indeed, as mentioned earlier, proven infection-related SUDI exhibit pathological findings identical to that observed in “classical” SIDS [as per the San Diego SIDS definition/category (1A)] (47). This review has postulated that the vagus might play an important role in modulating the immune response in such a way as to make babies with occult or overt infection more likely to die of sepsis. Neurophysiological and neurochemical responses to serious bacterial infection in infancy require further research to reveal possible specific effects of prone position on such physiological events that might increase mortality risk. Deutschman and Tracey (48) argue that currently understood immunopathological mechanisms involved in sepsis fail to adequately explain cellular dysfunction and organ failure and death. They call for new ideas in immunometabolics and neurophysiology to explain the dysfunction of endothelial and epithelial barriers, which lead to organ failure and death (48). New avenues of research should also help explain why sepsis is often difficult to diagnose in the living as it is postmortem. In a vulnerable host death from sepsis can be rapid and can be missed at autopsy. Rapid-onset sepsis and death may not leave the hallmarks of infection (49); and features of sepsis may not be apparent at autopsy except for bacteria in normally sterile sites in some cases. Markers of infection such as CD68 cells are not consistently found in the lungs of SIDS cases (1). This may reflect the often fulminant nature of the septic process before conscription of measurable numbers immune cells to be observed as a marker. Neonates who have died of confirmed sepsis show extremely variable innate and adaptive immune responses (50) probably reflecting immaturity of their immune system.

The true success of the “back-to-sleep” campaign in reducing SIDS deaths will be seen when further research is able to fully explain the fatal physiological events occurring in vulnerable infants. Infection (particularly with the key players *S. aureus* and *E. coli*) (8, 12, 13) interacting with vagal and/or spinal neural pathways concerned with neuroimmunological and neurochemical physiology and inflammatory responses in predisposed infants should provide a fruitful pathway for research (51). A complete explanation of SIDS etiopathogenesis should arise from this together with a deeper understanding of fatal perinatal infections.

## Author’s Note

Work pertaining to some sections of the paper was supported by the Foundation for the Study of Infant Death, United Kingdom.

## Author Contributions

The author confirms being the sole contributor of this work and approved it for publication.

## Conflict of Interest Statement

The author declares that the research was conducted in the absence of any commercial or financial relationships that could be construed as a potential conflict of interest.
